# Guanfacine monotherapy for ADHD/ASD comorbid with Tourette syndrome: a case report

**DOI:** 10.1186/s12991-019-0226-6

**Published:** 2019-03-11

**Authors:** Kosuke Okazaki, Kazuhiko Yamamuro, Junzo Iida, Toshifumi Kishimoto

**Affiliations:** 1 Department of Psychiatry, Nara Medical University School of Medicine, 840 Shijo-cho Kashihara, Nara, 634-8521 Japan; 2 Faculty of Nursing, Nara Medical University School of Medicine, Kashihara, Japan

**Keywords:** Guanfacine, Atomoxetine, Methylphenidate, Attention deficit/hyperactivity disorder, Autism spectrum disorder, Tourette syndrome

## Abstract

**Background:**

Patients with attention deficit/hyperactivity disorder (ADHD) often experience comorbid conditions, such as autism spectrum disorder (ASD) and Tourette syndrome (TS). Although pharmacotherapies are effective for treating ADHD, they are likely to elicit a variety of adverse effects. It is, thus, important to select an effective and well-tolerated pharmacotherapeutic treatment for patients with ADHD/ASD comorbid with TS.

**Case presentation:**

We report the case of a 10-year-old boy with ADHD/ASD comorbid with TS who was treated with guanfacine (GUAN). He experienced several side effects of atomoxetine (ATX) and methylphenidate (MPH) before being treated with GUAN. In the presented case, symptoms of ADHD as well as tic symptoms were improved by treatment with GUAN.

**Conclusion:**

GUAN might be effective and well tolerated in the treatment of patients with ADHD/ASD comorbid with TS who experience side effects of ATX and MPH.

## Background

Attention deficit/hyperactivity disorder (ADHD), which affects 5%–10% of school-aged children [1], is characterized by inattention, hyperactivity, impulsivity, and abnormalities in cognitive processes [2]. Recently, in the Diagnostic and Statistical Manual of Mental Disorders, 5th edition (DSM-5), the term “pervasive developmental disorders” (PDD) has been changed to “autism spectrum disorder” (ASD), and the coexistence of ADHD and ASD (ADHD/ASD) is now recognized. In a 2001 survey, 83% of children with PDD were also diagnosed with ADHD [3]. Similarly, in Japan, 67.9% of high-functioning children with PDD meet the diagnostic criteria for ADHD [4]. Also, many patients with ADHD suffer from comorbidities such as Tourette syndrome (TS) [5]. ADHD is mainly treated with non-pharmacological interventions and pharmacotherapy [6]. Pharmacotherapy, in particular, is important for the treatment of moderate to severe symptoms of ADHD in children and adolescents, including, for example, psychostimulant methylphenidate (MPH) and the selective norepinephrine (NE) reuptake inhibitor atomoxetine (ATX) [7]. MPH acts as an indirect dopamine (DA) agonist, inhibiting DA reuptake by occupying the DA transporter [8] and thus increasing DA in the striatum and the prefrontal cortex (PFC) [9]. In contrast, although ATX is a selective NE reuptake inhibitor, it also inhibits DA reuptake in the PFC. It has been found that NE transporters are relatively abundant compared with DA transporters in the PFC [10, 11]. In addition, it has been shown that DA is taken up non-selectively as well as co-released by NE transporters in the PFC [12–14]. The NE transporter has similar affinities for NE [15] and DA, and DA that is released extracellularly may diffuse transsynaptically to the NE transporters [13]. While this does not increase DA in the striatum, it increases both DA and NE in the PFC [9]. These findings suggest that both types of medication increase DA in the PFC. Approximately, 10%–30% of children and adolescents with ADHD are unresponsive to psychostimulant medications or experience adverse effects such as exacerbation of comorbid psychiatric disorders (tic symptoms), loss of appetite, insomnia, anxiety, and sympathomimetic cardiovascular side effects [16, 17]. Moreover, previous research has found that 18% of children with ASD/ADHD discontinued treatment due to adverse effects, particularly irritability, compared to only 1.4% of children with only ADHD [18].

TS, on the other hand, is characterized by the presence of multiple involuntary motor and vocal tics. TS is treated with pharmacological medications such as D2-DA receptor antagonists combined with psychological behavior therapy [19]. Importantly, pharmacological treatments for ADHD and TS have the opposite effect in the PFC.

Guanfacine (GUAN) was approved for children and adolescents (6–17 years) in Japan in May 2017. Orally administered GUAN is rapidly and completely absorbed, with maximum plasma concentrations occurring 1–4 h after administration [20]. The total clearance of GUAN from human plasma equals 11–22 Lh, and the drug is mainly metabolized by the liver, with 24–37% of GUAN from human plasma excreted unchanged by the kidneys [20]. The pharmacokinetics of GUAN are essentially identical in erythrocytes and the plasma. At an average hematocrit value of 32%, the distribution of GUAN is 60% in the plasma and 40% in erythrocytes [21]. It directly stimulates postsynaptic α_2A_-adrenergic receptors in the PFC to enhance noradrenaline neurotransmission [22], thereby strengthening the cortical network [23]. A beneficial effect of GUAN on ADHD core symptoms in children and adolescents with ADHD has been shown, without modulation of DA in the PFC [24].

To the best of our knowledge, this is the first case report that assesses the use of GUAN in the treatment of a patient with ADHD/ASD comorbid with TS. This case corroborates the effectiveness of GUAN as a viable alternative to other pharmacological treatments for patients with ADHD/ASD comorbid with TS.

The symptoms of the current case and the severity of ADHD were assessed using the Japanese version of the ADHD Rating Scale IV (ADHD-RS-IV-J) of the ADHD-RS-IV “home version”, which is an 18-item scale that is reliable and easy to administer. In addition, the tic severity of the current case was assessed using the Yale Global Tic Severity Scale (YGTSS), in which a higher YGTSS score is associated with higher tic symptom severity. This scale yields three summary scores: total motor (0–25), total phonic (0–25), and total tic (sum of motor and phonic) scores. The YGTSS also contains an impairment scale (0–50), which evaluates the global level of functional impairment arising from tics.

## Case presentation

The 10-year-old boy described here (Full Intelligence Quotient [FIQ] = 112, Verbal Intelligence Quotient [VIQ] = 106, Performance Intelligence Quotient [PIQ] = 117) had been diagnosed with a developmental delay in head control, speech, and language by a paediatrician when he was 1 year and 6 months old. When he entered kindergarten, he often played by himself and did not make friends because of his communication problems. After entering the local elementary school, at the age of 6, he began to show hyperactivity and impulsivity. In addition, he displayed symptoms of motor and vocal tics. He was assessed at a local clinic, and diagnosed with ADHD/ASD comorbid with TS. Although he initially continued to take risperidone (0.5 mg/day), side effects such as headache and anxiety led him to discontinue the treatment. When he was 9 years old, worsened impulsivity led him to behave violently toward his mother. He, therefore, began treatment, at a local clinic, with atomoxetine (ATX) (30 mg/day). However, he discontinued the medication as he experienced worsening irritability. Although he was prescribed MPH (18 mg/day) after discontinuing the treatment with ATX, he also discontinued taking MPH, because his motor and vocal tic symptoms were exacerbated. As these symptoms continued, he was referred to our hospital at 10 years of age, with an ADHD-RS-IV-J score of 23 and a YGTSS score of 29.

According to his father, he had only few friends because he had so little interest in making friends in school. The teachers often reported problems to his parents, such as when he showed physical aggression toward his friends or ran away from school during the lesson. He often got angry when the timing of activities deviated from his usual schedule. He was, therefore, diagnosed with ADHD/ASD/TS according to the criteria specified in the DSM-5.

**Fig. 1:**
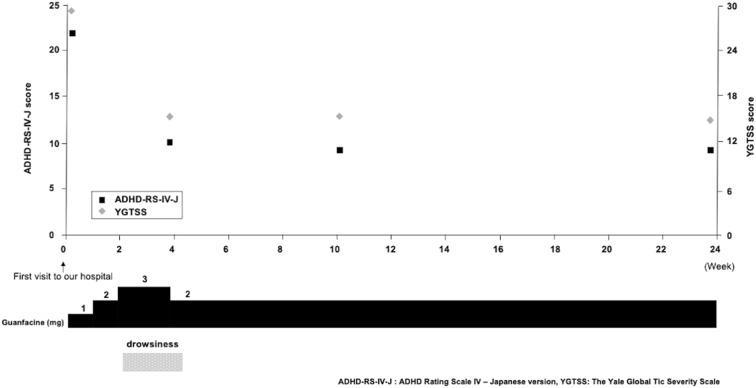
Course of the ADHD-RS-IV-J score and YGTSS score

The patient was continuously prescribed GUAN at a dose starting at 1 mg/day and increasing to 3 mg/day. However, the 3-mg dose led to drowsiness (ADHD-RS-IV-J score of 10, YGTSS score of 15), and was, therefore, reduced again to 2 mg/day. At the decreased dose, he continued to take GUAN without side effects (ADHD-RS-IV-J score of 9, YGTSS score of 15), while there was no clear difference in effect between the 2-mg/day and the 3-mg/day doses. Importantly, his ADHD-related symptoms, such as irritability, hyperactivity, and inattention, as well as his tic symptoms, gradually improved. On the other hand, GUAN had no effect on ASD symptoms in this case. The patient was able to continue taking GUAN for 6 more months (ADHD-RS-IV-J score of 9, YGTSS score of 15) (Fig. 1).

## Discussion

To the best of our knowledge, the case presented here represents original evidence for the effectiveness of GUAN monotherapy and shows that it is well tolerated and improves ADHD symptoms and tic symptoms in a patient with ADHD/ASD comorbid with TS.

Psychostimulant and non-psychostimulant medications, such as MPH and ATX, are currently the first-choice medication for children and adolescents with ADHD [7]. Psychostimulants, therefore, are tried for the treatment of many patients with ADHD, and approximately 70% of children and adolescents with ADHD respond to a single stimulant [25]. However, previous reports showed that psychostimulants could cause exacerbation of tic symptoms in patients with ADHD comorbid with TS [26]. A pathogenic hypothesis of tics associates them with increased DA activity in the basal ganglia [27], while dysfunction in dopaminergic fronto-striatal neuronal networks causes dysfunction of inhibitory executive functions or cognitive control [28]. Psychostimulants are blocking the DA transporters in the synapse and increase extracellular DA levels in the brain [29]. In consequence, they are effective for patients with ADHD but could exacerbate tic symptoms. In contrast to MPH, GUAN stimulates post-synaptic α_2A_-adrenergic receptors and enhances noradrenaline neurotransmission [22, 30, 31]. Several studies have shown reliable effects of GUAN, as monotherapy or as adjunctive therapy with stimulants, in the treatment of children and adolescents with ADHD [24, 32]. Interestingly, α_2_ adrenergic receptor agonists, such as clonidine, are effective for patients with TS [33]. We, therefore, suggest that GUAN is effective as well as clonidine, for both patients with ADHD and those with TS. Furthermore, previous studies have revealed that GUAN has beneficial effects in the treatment of patients with ADHD comorbid with TS [34, 35].

The present study shows that GUAN improved tic as well as ADHD symptoms in a patient with ADHD/ASD comorbid with TS, although MPH caused an exacerbation of tic symptoms. This case indicates that treatment with GUAN is effective and well tolerated in patients with ADHD/ASD comorbid with TS. However, GUAN led to drowsiness in the current case. A previous study showed that the most common side effects are somnolence (30.4%) and sedation (13.3%) [36], as the α_2_-adrenergic receptor agonist leads to high peak plasma concentrations associated with these side effects [37, 38]. The side effects were, however, improved by decreasing the GUAN dose.

No previous study has shown that GUAN influences ASD symptoms, and our case likewise showed no effect on ASD symptoms. GUAN might, thus, be an alternative treatment for some patients who experience side effects of ATX and MPH, without exacerbating other psychiatric symptoms.

The main limitation of our study is that it involves only one case, and we should, therefore, be careful when drawing conclusions. Future studies are, therefore, needed to continuously assess the effectiveness of GUAN in treating patients with ADHD comorbid with ASD and TS.

## Conclusion

This case indicates that GUAN might be an efficacious and well-tolerated treatment for ADHD and tic symptoms in patients with ADHD/ASD comorbid with TS, because pharmacological medication tends to cause different side effects in patients with ADHD comorbid with ASD than in those with only ADHD or ASD. Further studies with larger sample sizes are needed to validate the apparent efficacy, safety, and tolerability of medical treatment with GUAN.

## Abbreviations

ADHD: attention deficit/hyperactivity disorder
ADHD-RS-IV-J: ADHD Rating Scale IV-Japanese version
ASD: autism spectrum disorder
ATX: atomoxetine
FIQ: full intelligence quotient
GUAN: guanfacine
MPH: methylphenidate
OCD: obsessive–compulsive disorder
ODD: oppositional defiant disorder
PDD: pervasive developmental disorders
PIQ: performance intelligence quotient
PO: perceptual organization
PS: process speed
TS: Tourette syndrome
VC: verbal comprehension
VIQ: verbal intelligence quotient
YGTSS: Yale Global Tic Severity Scale
